# Features of Human Herpesvirus-6A and -6B Entry

**DOI:** 10.1155/2012/384069

**Published:** 2012-10-23

**Authors:** Takahiro Maeki, Yasuko Mori

**Affiliations:** ^1^Division of Clinical Virology, Kobe University Graduate School of Medicine, Kusunoki-cho, Chuo-ku, Kobe 650-0017, Japan; ^2^Laboratory of Virology and Vaccinology, Division of Biomedical Research, National Institute of Biomedical Innovation, 7-6-8 Saito-Asagi, Ibaraki, Osaka 567-0085, Japan

## Abstract

Human herpesvirus-6 (HHV-6) is a T lymphotropic herpesvirus belonging to the *Betaherpesvirinae* subfamily. HHV-6 was long classified into variants A and B (HHV-6A and HHV-6B); however, recently, HHV-6A and HHV-6B were reclassified as different species. The process of herpesvirus entry into target cells is complicated, and in the case of HHV-6A and HHV-6B, the detailed mechanism remains to be elucidated, although both viruses are known to enter cells via endocytosis. In this paper, (1) findings about the cellular receptor and its ligand for HHV-6A and HHV-6B are summarized, and (2) a schematic model of HHV-6A's replication cycle, including its entry, is presented. In addition, (3) reports showing the importance of lipids in both the HHV-6A envelope and target-cell membrane for viral entry are reviewed, and (4) glycoproteins involved in cell fusion are discussed.

## 1. Introduction

 The *herpesviridae* are a family of double-stranded enveloped DNA viruses. Their entry into host cells proceeds as follows. First, the virus binds to its target cell through a specific receptor. Second, herpesviruses enter cells via two different pathways: (a) direct fusion of the viral envelope with the target-cell plasma membrane or (b) endocytosis followed by fusion between the viral and cellular membranes in the endosomal compartment [1]. 

Human herpesvirus-6 (HHV-6) was initially isolated from the peripheral blood of patients with lymphoproliferative disorders, in 1986 [2]. It belongs to the *Betaherpesvirinae *subfamily, along with human cytomegalovirus (HCMV) and Human herpesvirus-7 (HHV-7), and is a member of the genus *Roseolovirus*, along with HHV-7. HHV-6 was originally classified into variants A and B (HHV-6A and HHV-6B), based on differences in genetic, antigenic, and growth characteristics [3–5]. However, recently, HHV-6A and HHV-6B were reclassified into different species (Virus Taxonomy List 2011). The homology of entire genome sequence between both is nearly 90% [6–8]. Primary infection of HHV-6B causes exanthem subitum [9], and HHV-6A has been reported to be involved in several diseases, including encephalitis [10], hepatitis [11], glioma [12], and multiple sclerosis [13].

However, the detailed replication cycle of HHV-6A and HHV-6B after entering the cell remains to be elucidated. For some of the steps, different groups have reported conflicting results.

 Regarding the ligand and receptor for HHV-6A and HHV-6B, Santoro et al. reported that the cellular receptor for both viruses is CD46 [14]. Our group showed that the glycoprotein gH/gL/gQ1/gQ2 complex [15] is the ligand for HHV-6A [16], and that its receptor is CD46 [17, 18]. However, in the case of HHV-6B (strain HST, an isolate from an infant with exanthem subitum), we reported that, of two complexes found in this virus, gH/gL/gQ and gH/gL/gO neither binds to CD46 [19]. This discrepancy might be due to the difference in HHV-6B strain (Santoro et al. used strains Z29 and PL1, while we used HST) or some other reason.

In this paper, (1) previous reports about the cellular receptor and its ligand for HHV-6A and HHV-6B are summarized, and (2) findings about the entry of HHV-6A and HHV-6B into host cells are reviewed, and a schematic model of HHV-6A's replication cycle is presented. In addition, (3) reports showing the importance of lipids in both the HHV-6A envelope and target-cell membrane for viral entry are reviewed. Finally, (4) glycoproteins that have been shown to play a critical role in cell fusion (glycoprotein H, glycoprotein B) are discussed briefly.

## 2. The Cellular Receptor and Its Ligand for HHV-6A and HHV-6B

### 2.1. HHV-6A

 As described above, Santoro et al. reported that HHV-6A and HHV-6B use the human complement regulator CD46 as a cellular receptor [14], and our group showed that the gH/gL/gQ1/gQ2 complex of HHV-6A binds to CD46 [17]. Santoro et al. identified gH as the CD46-binding component of HHV-6A. They showed that (i) an anti-CD46 antibody immunoprecipitated gH in HHV-6A- (GS-strain-) infected cells, (ii) the anti-CD46 antibody did not immunoprecipitate gH from gH-depleted HHV-6A-infected cells, although it did immunoprecipitate gH from gp82-105- or gB-depleted HHV-6A-infected cells, and (iii) a specific anti-gH antibody immunoprecipitated CD46 in HHV-6A-infected cells [20].

 Our group showed that not only an anti-gH antibody but also an anti-gQ1 antibody could immunoprecipitate CD46 from HHV-6A- (GS-strain-) infected cells [18]. Furthermore, we recently confirmed that an anti-gQ1 monoclonal antibody (Mab) immunoprecipitates CD46 from HHV-6A- (GS-strain-) infected cells (unpublished data) and showed that CD46immunoprecipitates gH, gL, gQ1, and gQ2 from cells transfected with all four genes [17]. We also showed that the maturation of gQ1, which is indicated by its size shift from 74 kDa (gQ1-74 K) to 80 kDa (gQ1-80 K), occurs only when all four components, gH, gL, gQ1, and gQ2, are coexpressed, and that gQ1-80 K, but not gQ1-74 K, contributes to the gH/gL/gQ1/gQ2 complex and binds to CD46 [17]. 

 CD46 is a member of the glycoprotein family called regulators of complement activation (RCA) [21, 22]. CD46 is a type I transmembrane glycoprotein of 45–67 kDa expressed on all nucleated cells [23]. CD46 contains four short consensus repeats (SCRs) in its N-terminal region, followed by a serine-threonine-proline (STP) rich domain, a small region of unknown significance, a transmembrane domain, and a cytoplasmic tail. Four distinct CD46 isoforms generated by alternative RNA splicing are expressed differentially in various cell types; these isomers have the same SCRs but different STP or cytoplasmic domains [21]. CD46 functions as a cofactor in the factor-I-mediated proteolytic cleavage of C3b and C4b; this process protects host cells from inadvertent lysis by the complement system. CD46's interactions with C3b and C4b involve several regions on SCR2, SCR3, and SCR4 [24]. CD46 was recently reported to have roles not only in innate immunity but also in adaptive immunity [23]. Furthermore, it was demonstrated that CD46 induces autophagy upon pathogen recognition [25]. 

 CD46 is also used as an entry receptor for several human viruses and bacteria. SCR1 and SCR2 are critical domains for measles virus, SCR3 and the STP region for Neisseria gonorrhoeae, and SCR3 and SCR4 for group A streptococcus [24]. For HHV-6A, our group showed that SCR2, SCR3, and SCR4 are required for virus-mediated cell-cell fusion [26], although other groups reported that SCR2 and SCR3 are the critical determinants for CD46 binding [20] and cell fusion [27]. The reason for the discrepancy is uncertain. It is possible that the direct binding domains are in SCR2 and SCR3, while SCR4 is required only to maintain the conformation of the binding site, because Santoro et al. replaced the SCR4 of CD46 with that of DAF (decay accelerating factor), whereas we deleted SCR4. Further investigation is required to clarify this issue. The structure of extracellular portion of CD46 was recently reported [24], but that of its HHV-6A ligand, the gH/gL/gQ1/gQ2 complex, still needs to be determined.

 Of the four components of the HHV-6A gH/gL/gQ1/gQ2 complex, our group reported that gQ1 and gQ2 are essential for viral infection [17, 28]. We also showed that, in addition to gH/gL/gQ1/gQ2, the HHV-6A viral envelope contains the complex gH/gL/gO, which does not bind to human CD46 [19]. The specific molecular function of HHV-6A gO and the gH/gL/gO complex remains to be elucidated. In HCMV, the gH/gL/gO complex is necessary for viral entry into human fibroblasts [29]. In addition, in EBV, the gH/gL complex associates with gp42, and this gH/gL/gp42 complex is necessary for viral entry into B cells but not into epithelial cells [30]. CMV also encodes glycoproteins that redirect cell tropism by forming complexes with gH/gL [31]. The predicted amino acid identity between the gO of HHV-6A and HHV-6B is 76.8%, which is much lower than that of other glycoproteins. Therefore, the gH/gL/gO complex may confer different biological properties in HHV-6A and HHV-6B, including cell tropism. Efforts to elucidate the function of gO and gH/gL/gO are underway. 

### 2.2. HHV-6B

 In 1999, Santoro et al. reported that CD46 is the receptor for both HHV-6A and HHV-6B [14]. They showed that (i) an anti-CD46 antibody inhibited HHV-6B (strain Z29) infection and HHV-6B-mediated cell fusion in PBMCs and (ii) the expression of CD46 in NIH3T3 cells (mouse fibroblasts) and EL4 cells (murine T lymphoblasts) caused HHV-6B- (strain-PL1-) mediated fusion and entry, respectively. In addition, Pedersen et al. reported that HHV-6B (strain PL1) causes fusion from without (FFWO) in HEK293 and SupT-1 cells [32, 33]. 

 However, our group showed that the HHV-6B (strain HST) virion contains gH/gL/gO and gH/gL/gQ complexes, but that neither of them binds to CD46 [19], and that HHV-6B (strain HST) does not mediate FFWO in various cell types expressing human CD46, except for MT4 cells [26]. The discrepancy between these results could be attributable to several differences, including the titer of HHV-6B infection, and the HHV-6B virus strain. 

Regarding the glycoprotein complex, it has been found that HHV-6B gH/gL/gQ1/gQ2 complex also plays an important role for the entry [34].

## 3. Replication Cycle of HHV-6A and HHV-6B

### 3.1. HHV-6A

 As shown above, entry of herpesviruses into cells occurs in two distinct steps. In 1992, Cirone et al. showed that HHV-6A (strain GS) enters the T-lymphoblastoid cell line, HSB-2 cells, via endocytosis and that no fusion event occurs at the plasma membrane [35]. Since then, no other reports have been shown regarding the HHV-6 entry step. For HHV-6A assembly, the envelopment-deenvelopment-reenvelopment pathway has been proposed [36, 37]. In this model, HHV-6A assembly occurs as follows. (i) The intranuclear naked capsid (around 80 nm in diameter) buds into the perinuclear cisternae, and the nucleocapsid acquires a primary envelope, which is devoid of glycoprotein. (ii) Deenvelopment of the nucleocapsid occurs in the cytoplasm, as shown by the presence of cytoplasmic naked nucleocapsids (around 140 nm in diameter). (iii) The naked nucleocapsid again acquires an envelope as well as spikes in cytoplasmic vesicles, and, finally, mature viral particles (around 185 nm in diameter) form. However, many details of this process remain to be elucidated. 

Different models have been proposed for tegumentation and for the compartment in which HHV-6A reenvelopment occurs. Regarding tegument acquisition, Roffman et al. reported that HHV-6B (Z29 strain) virions in infected thymocytes acquire their tegument in tegusomes, which are spherical intranuclear compartments resulting from cytoplasmic invagination into the nucleus, because they contain ribosomes [38]. However, Torrisi et al. could not find such structures in HHV-6A strain GS-infected HSB-2 cells [36]. Later, Ahlqvist et al. suggested that tegumentation of HHV-6A can occur in the nucleus, in the tegusome or in the cytoplasm, or in either of the compartment from the analysis with U1102 infected SupT-1, lymphoblastoid cell line, and HPDA(human progenitor-derived astrocytes) [37]. The different results obtained in these reports could be due to differences in the virus (HHV-6A or HHV-6B), viral strain (HHV-6A GS or U1102), or type of cells used.

 As to the reenvelopment compartment, since viral glycoproteins (gB and gH) are absent from the HHV-6A- (strain-GS-) infected HSB-2 cell plasma membrane, the plasma membrane is unlikely to be the site for reenvelopment [39, 40]. Torrisi et al. suggested that reenvelopment at annulate lamellae (AL) is required for HHV-6 to acquire an envelope with spike protein [36]. Cardinali et al. reported that HHV-6A- (strain-GS-) infection induced AL in HSB-2 cells, and proposed the AL as a putative site for oligosaccharide addition, based on the results of labeling with HPL (Helix pomatia lectin, which recognizes intermediate forms of glycoconjugates after the O-linked addition of sugar in cis-Golgi cisternae) and WGA (Wheat germ agglutinin, which binds terminally glycosylated components) [41]. However, Ahlqvist reported that no AL were found in HHV-6A- (strain-U1102-) infected astrocytes (HPDA), and that although a low percentage of U1102-infected SupT-1 cells had AL, no viral particles could be found in them. The authors suggested that the spikes of mature viral particles are acquired at cytoplasmic vesicles of unknown origin [37].

 Recently, our group showed that HHV-6A (strain GS) induces MVB (multivesicular body) formation, that the final envelopment of HHV-6A occurs at trans-Golgi network (TGN-) or post-TGN-derived membranes, and that enveloped virions are released by the exosomal pathway [42]. We found AL structures in HHV-6A-infected cells and some virions inside AL structures. However, the membranes that surrounded or enwrapped the HHV-6A virions were not derived from AL, because the enveloped capsids in the AL were found inside vacuoles that were further enwrapped by AL membrane. Furthermore, immunostaining with anti-gB and anti-gM antibodies showed that gB and gM were abundant on the nucleocapsid-enwrapping membranes, while they were scarce on the AL, suggesting that the origins of the two membranes were different. Regarding tegumentation, we found tegument-like electron-dense material on the cytosolic side of TGN-derived vacuoles and at other regions on these vacuoles.

**Figure 1:**
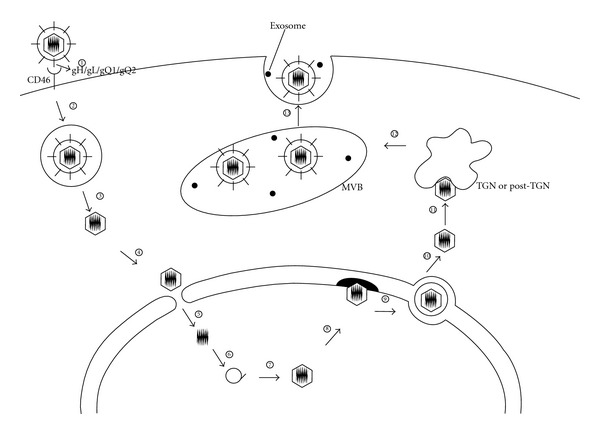
Schematic representation of the HHV-6A replication cycle. A diagram of the proposed replication cycle for HHV-6A is shown. HHV-6A gH/gL/gQ1/gQ2 binds to CD46 (1) and enters the cell via endocytosis (2). The deenveloped nucleocapsid is then transported to the nucleus (3, 4), where the viral genome is released (5). After viral gene transcription and genome replication (6), the progeny DNA is encapsidated (7), and the capsid buds into the perinuclear cisternae (8). The capsid acquires a primary envelope in the perinuclear space (9), and deenvelopment occurs in the cytoplasm (10). The nucleocapsid acquires the final envelope by budding into TGN- or post-TGN-derived membrane (11). Vacuoles containing virions expand and form MVBs (12), then the mature enveloped virions are released via the exosomal pathway (13).

 A model for the replication cycle of HHV-6A based on published reports is as follows [43] (Figure 1). The HHV-6A ligand, the gH/gL/gQ1/gQ2 complex, binds to its receptor CD46 (1), the other viral glycoprotein(s) (e.g., gB) also binds to unidentified cellular receptor(s) and enters the cell via endocytosis (2). After deenvelopment by fusion between the viral and cellular membranes in the endosomal compartment (3), the incoming nucleocapsid is transported through the cytoplasm to the nuclear pore complex (4), where the viral DNA genome is unpackaged and released into the nucleus (5). In the nucleus, viral gene transcription and genome replication occur (6). Long concatemeric strands of progeny DNA are cleaved to unit lengths and encapsidated (7). The capsids bud into the perinuclear cisternae (8) and acquire a primary envelope in the perinuclear space (9). Deenvelopment of the nucleocapsid occurs in the cytoplasm (10), and reenvelopment occurs by budding into TGN- or post-TGN-derived membranes (11). Finally, the virion-containing vacuoles expand and MVBs are formed (12), and the enveloped virions are released by the exosomal pathway by fusion of the MVBs with the plasma membrane (13).

### 3.2. HHV-6B

 Compared with HHV-6A, fewer reports have been published on the details of HHV-6B replication. In 1990, Nii et al. proposed the envelopment-deenvelopment-reenvelopment pathway model for HHV-6B (Hashimoto), based on examinations of infected MT4 cells and human lymphocytes [44]. Ahlqvist et al. supported this model with observations in Z29-infected SupT-1 cells [37].

 Conflicting results had been reported about HHV-6B's tegumentation, as with HHV-6A. Nii et al. found that tegument-coated capsids could be detected only in the cytoplasm, and not in the nucleus or perinuclear cisternae, suggesting that tegumentation occurred in the cytoplasm [44]. However, as described above, Roffman et al. reported that HHV-6B (Z29 stain) acquires its tegument at the tegument compartment [38], and Ahlqvist et al. supported this model by showing that Z29-infected SupT-1 cells form tegusomes [37]. Regarding AL, Ahlqvist et al. reported that no AL could be found in the Z29-infected SupT-1 cells [37]. 

## 4. Lipid in the HHV-6A Envelope and Target-Cell Membrane

 Our group showed that cholesterol in both the HHV-6A envelope and target cell is required for HHV-6A entry [45, 46]. In addition, we showed that HHV-6A infection induces the relocation of CD46 into lipid rafts and that glycoproteins (gQ1 and gB) are associated with lipid rafts, indicating that lipid rafts of the cell membrane are important for viral entry and that HHV-6A may enter the target cells via lipid rafts [46]. Furthermore, we reported that the HHV-6A envelope contains lipid rafts, suggesting that HHV-6A virions might assemble through lipid rafts [47]. These results show that lipid rafts in both the HHV-6A envelope and its target cells play a critical role in HHV-6A entry and might be involved in HHV-6A virus assembly.

## 5. Glycoproteins That May Be Involved in Cell Fusion

 Glycoprotein B (gB), glycoprotein H (gH), and glycoprotein L (gL), are conserved in all herpesviruses, and essential for entry [48]. As in other herpesviruses, gB and gH in HHV-6A and HHV-6B are known targets for neutralizing antibodies, so they are known to be major determinants for cell entry in HHV-6A and HHV-6B [49, 50]. Futhermore, we showed that gB and gH are required for U1102-induced polykaryocyte formation [26]. 

For other herpesviruses, especially HSV, reports have accumulated about the roles of viral glycoproteins in entry [1, 29, 30, 51–53], and structural analyses of herpesvirus glycoproteins have been performed [54–56]. EBV utilizes gp42 with gH/gL as a switch of cell tropism and the structural analysis of gp42 has also been done [1, 57]. 

However, in the case of HHV-6A and HHV-6B, the structures of the gH/gL or gH/gL/gQ1/gQ2 complexes have not yet been reported, and the detailed mechanisms remain unknown. To unveil the entry mechanisms in detail, determination of the gH/gL and gH/gL/gQ1/gQ2 structures and detailed analyses of each glycoprotein's function are needed.

## References

[1] Connolly SA, Jackson JO, Jardetzky TS, Longnecker R (2011). Fusing structure and function: a structural view of the herpesvirus entry machinery. *Nature Reviews Microbiology*.

[2] Salahuddin SZ, Ablashi DV, Markham PD (1986). Isolation of a new virus, HBLV, in patients with lymphoproliferative disorders. *Science*.

[3] Ablashi DV, Balachandran N, Josephs SF (1991). Genomic polymorphism, growth properties, and immunologic variations in human herpesvirus-6 isolates. *Virology*.

[4] Aubin JT, Collandre H, Candotti D (1991). Several groups among human herpesvirus 6 strains can be distinguished by Southern blotting and polymerase chain reaction. *Journal of Clinical Microbiology*.

[5] Chandran B, Tirawatnapong S, Pfeiffer B, Ablashi DV (1992). Antigenic relationships among human herpesvirus-6 isolates. *Journal of Medical Virology*.

[6] Isegawa Y, Mukai T, Nakano K (1999). Comparison of the complete DNA sequences of human herpesvirus 6 variants A and B. *Journal of Virology*.

[7] Gompels UA, Nicholas J, Lawrence G (1995). The DNA sequence of human herpesvirus-6: structure, coding content, and genome evolution. *Virology*.

[8] Dominguez G, Dambaugh TR, Stamey FR, Dewhurst S, Inoue N, Pellett PE (1999). Human herpesvirus 6B genome sequence: coding content and comparison with human herpesvirus 6A. *Journal of Virology*.

[9] Yamanishi K, Okuno T, Shiraki K (1988). Identification of human herpesvirus-6 as a causal agent for exanthem subitum. *Lancet*.

[10] Portolani M, Pecorari M, Tamassia MG, Gennari W, Beretti F, Guaraldi G (2001). Case of fatal encephalitis by HHV-6 variant A. *Journal of Medical Virology*.

[11] Potenza L, Luppi M, Barozzi P (2008). HHV-6A in syncytial giant-cell hepatitis. *New England Journal of Medicine*.

[12] Chi J, Gu B, Zhang C (2012). Human herpesvirus 6 latent infection in patients with glioma. *Journal of Infectious Diseases*.

[13] Akhyani N, Berti R, Brennan MB (2000). Tissue distribution and variant characterization of human herpesvirus (HHV)-6: increased prevalence of HHV-6A in patients with multiple sclerosis. *Journal of Infectious Diseases*.

[14] Santoro F, Kennedy PE, Locatelli G, Malnati MS, Berger EA, Lusso P (1999). CD46 is a cellular receptor for human herpesvirus 6. *Cell*.

[15] Akkapaiboon P, Mori Y, Sadaoka T, Yonemoto S, Yamanishi K (2004). Intracellular processing of human herpesvirus 6 glycoproteins Q1 and Q2 into tetrameric complexes expressed on the viral envelope. *Journal of Virology*.

[16] Mori Y (2009). Recent topics related to human herpesvirus 6 cell tropism. *Cellular Microbiology*.

[17] Tang H, Hayashi M, Maeki T, Yamanishi K, Mori Y (2011). Human herpesvirus 6 glycoprotein complex formation is required for folding and trafficking of the gH/gL/gQ1/gQ2 complex and its cellular receptor binding. *Journal of Virology*.

[18] Mori Y, Yang X, Akkapaiboon P, Okuno T, Yamanishi K (2003). Human herpesvirus 6 variant A glycoprotein H-glycoprotein L-glycoprotein Q complex associates with human CD46. *Journal of Virology*.

[19] Mori Y, Akkapaiboon P, Yonemoto S (2004). Discovery of a second form of tripartite complex containing gH-gL of human herpesvirus 6 and observations on CD46. *Journal of Virology*.

[20] Santoro F, Greenstone HL, Insinga A (2003). Interaction of glycoprotein H of human herpesvirus 6 with the cellular receptor CD46. *Journal of Biological Chemistry*.

[21] Seya T, Hirano A, Matsumoto M, Nomura M, Ueda S (1999). Human membrane cofactor protein (MCP, CD46): multiple isoforms and functions. *International Journal of Biochemistry and Cell Biology*.

[22] Thulke S, Radonić A, Nitsche A, Siegert W (2006). Quantitative expression analysis of HHV-6 cell receptor CD46 on cells of human cord blood, peripheral blood and G-CSF mobilised leukapheresis cells. *Virology Journal*.

[23] Cardone J, Le Friec G, Kemper C (2011). CD46 in innate and adaptive immunity: an update. *Clinical and Experimental Immunology*.

[24] Persson BD, Schmitz NB, Santiago C (2010). Structure of the extracellular portion of CD46 provides insights into its interactions with complement proteins and pathogens. *PLoS Pathogens*.

[25] Joubert PE, Meiffren G, Grégoire IP (2009). Autophagy induction by the pathogen receptor CD46. *Cell Host and Microbe*.

[26] Mori Y, Seya T, Huang HL, Akkapaiboon P, Dhepakson P, Yamanishi K (2002). Human herpesvirus 6 variant A but not variant B induces fusion from without in a variety of human cells through a human herpesvirus 6 entry receptor, CD46. *Journal of Virology*.

[27] Greenstone HL, Santoro F, Lusso P, Berger EA (2002). Human herpesvirus 6 and measles virus employ distinct CD46 domains for receptor function. *Journal of Biological Chemistry*.

[28] Tang H, Kawabata A, Yoshida M (2010). Human herpesvirus 6 encoded glycoprotein Q1 gene is essential for virus growth. *Virology*.

[29] Revello MG, Gerna G (2010). Human cytomegalovirus tropism for endothelial/epithelial cells: scientific background and clinical implications. *Reviews in Medical Virology*.

[30] Hutt-Fletcher LM (2007). Epstein-Barr virus entry. *Journal of Virology*.

[31] Wang D, Shenk T (2005). Human cytomegalovirus virion protein complex required for epithelial and endothelial cell tropism. *Proceedings of the National Academy of Sciences of the United States of America*.

[32] Pedersen SM, Øster B, Bundgaard B, Höllsberg P (2006). Induction of cell-cell fusion from without by human herpesvirus 6B. *Journal of Virology*.

[33] Pedersen SM, Höllsberg P (2006). Complexities in human herpesvirus-6A and -6B binding to host cells. *Virology*.

[34] Kawabata A, Oyaizu H, Maeki T, Tang H, Yamanishi K, Mori Y (2011). Analysis of a neutralizing antibody for human herpesvirus 6B reveals a role for glycoprotein Q1 in viral entry. *Journal of Virology*.

[35] Cirone M, Zompetta C, Angeloni A (1992). Infection by human herpesvirus 6 (HHV-6) of human lymphoid T cells occurs through an endocytic pathway. *AIDS Research and Human Retroviruses*.

[36] Torrisi MR, Gentile M, Cardinali G (1999). Intracellular transport and maturation pathway of human herpesvirus 6. *Virology*.

[37] Ahlqvist J, Donati D, Martinelli E (2006). Complete replication cycle and acquisition of tegument in nucleus of human herpesvirus 6A in astrocytes and in T-cells. *Journal of Medical Virology*.

[38] Roffman E, Albert JP, Goff JP, Frenkel N (1990). Putative site for the acquisition of human herpesvirus 6 virion tegument. *Journal of Virology*.

[39] Cirone M, Campadelli-Fiume G, Foa-Tomasi L, Torrisi MR, Faggioni A (1994). Human herpesvirus 6 envelope glycoproteins B and H-L complex are undetectable on the plasma membrane of infected lymphocytes. *AIDS Research and Human Retroviruses*.

[40] Biberfeld P, Kramarsky B, Salahuddin SZ, Gallo RC (1987). Ultrastructural characterization of a new human B lymphotropic DNA virus (human herpesvirus 6) isolated from patients with lymphoproliferative disease. *Journal of the National Cancer Institute*.

[41] Cardinali G, Gentile M, Cirone M (1998). Viral glycoproteins accumulate in newly formed annulate lamellae following infection of lymphoid cells by human herpesvirus 6. *Journal of Virology*.

[42] Mori Y, Koike M, Moriishi E (2008). Human herpesvirus-6 induces MVB formation, and virus egress occurs by an exosomal release pathway. *Traffic*.

[43] De Bolle L, Naesens L, De Clercq E (2005). Update on human herpesvirus 6 biology, clinical features, and therapy. *Clinical Microbiology Reviews*.

[44] Nii S, Yoshida M, Uno F, Kurata T, Ikuta K, Yamanishi K (1990). Replication of human herpesvirus 6 (HHV-6): morphological aspects. *Advances in Experimental Medicine and Biology*.

[45] Huang H, Li Y, Sadaoka T (2006). Human herpesvirus 6 envelope cholesterol is required for virus entry. *Journal of General Virology*.

[46] Tang H, Kawabata A, Takemoto M, Yamanishi K, Mori Y (2008). Human herpesvirus-6 infection induces the reorganization of membrane microdomains in target cells, which are required for virus entry. *Virology*.

[47] Kawabata A, Tang H, Huang H, Yamanishi K, Mori Y (2009). Y Human herpesvirus 6 envelope components enriched in lipid rafts: evidence for virion-associated lipid rafts. *Virology Journal*.

[48] Spear PG, Longnecker R (2003). Herpesvirus entry: an update. *Journal of Virology*.

[49] Takeda K, Okuno T, Isegawa Y, Yamanishi K (1996). Identification of a variant A-specific neutralizing epitope on glycoprotein B (gB) of human herpesvirus-6 (HHV-6). *Virology*.

[50] Takeda K, Haque M, Sunagawa T, Okuno T, Isegawa Y, Yamanishi K (1997). Identification of a variant B-specific neutralizing epitope on glycoprotein H of human herpesvirus-6. *Journal of General Virology*.

[51] Eisenberg RJ, Atanasiu D, Cairns TM, Gallagher JR, Krummenacher C, Cohen GH (20124). Herpes virus fusion and entry: a story with many characters. *Viruses*.

[52] Atanasiu D, Saw WT, Cohen GH, Eisenberg RJ (2010). Cascade of events governing cell-cell fusion induced by herpes simplex virus glycoproteins gD, gH/gL, and gB. *Journal of Virology*.

[53] Campadelli-Fiume G, Amasio M, Avitabile E (2007). The multipartite system that mediates entry of herpes simplex virus into the cell. *Reviews in Medical Virology*.

[54] Chowdary TK, Cairns TM, Atanasiu D, Cohen GH, Eisenberg RJ, Heldwein EE (2010). Crystal structure of the conserved herpesvirus fusion regulator complex gH-gL. *Nature Structural and Molecular Biology*.

[55] Heldwein EE, Lou H, Bender FC, Cohen GH, Eisenberg RJ, Harrison SC (2006). Crystal structure of glycoprotein B from herpes simplex virus 1. *Science*.

[56] Matsuura H, Kirschner AN, Longnecker R, Jardetzky TS (2010). Crystal structure of the Epstein-Barr virus (EBV) glycoprotein H/glycoprotein L (gH/gL) complex. *Proceedings of the National Academy of Sciences of the United States of America*.

[57] Borza CM, Hutt-Fletcher LM (2002). Alternate replication in B cells and epithelial cells switches tropism of Epstein-Barr virus. *Nature Medicine*.

